# Shedding light on the molecular and regulatory mechanisms of TLR4 signaling in endothelial cells under physiological and inflamed conditions

**DOI:** 10.3389/fimmu.2023.1264889

**Published:** 2023-11-24

**Authors:** Anna Stierschneider, Christoph Wiesner

**Affiliations:** Department Science & Technology, Institute Biotechnology, IMC Krems University of Applied Sciences, Krems, Austria

**Keywords:** toll-like receptor 4 signaling, endothelium, pro-inflammatory response, lipopolysaccharide, optogenetic control

## Abstract

Toll-like receptor 4 (TLR4) are part of the innate immune system. They are capable of recognizing pathogen-associated molecular patterns (PAMPS) of microbes, and damage-associated molecular patterns (DAMPs) of damaged tissues. Activation of TLR4 initiates downstream signaling pathways that trigger the secretion of cytokines, type I interferons, and other pro-inflammatory mediators that are necessary for an immediate immune response. However, the systemic release of pro-inflammatory proteins is a powerful driver of acute and chronic inflammatory responses. Over the past decades, immense progress has been made in clarifying the molecular and regulatory mechanisms of TLR4 signaling in inflammation. However, the most common strategies used to study TLR4 signaling rely on genetic manipulation of the TLR4 or the treatment with agonists such as lipopolysaccharide (LPS) derived from the outer membrane of Gram-negative bacteria, which are often associated with the generation of irreversible phenotypes in the target cells or unintended cytotoxicity and signaling crosstalk due to off-target or pleiotropic effects. Here, optogenetics offers an alternative strategy to control and monitor cellular signaling in an unprecedented spatiotemporally precise, dose-dependent, and non-invasive manner. This review provides an overview of the structure, function and signaling pathways of the TLR4 and its fundamental role in endothelial cells under physiological and inflammatory conditions, as well as the advances in TLR4 modulation strategies.

## 1 Structure and function of the toll-like receptor 4 and its receptor family

**Figure 1:**
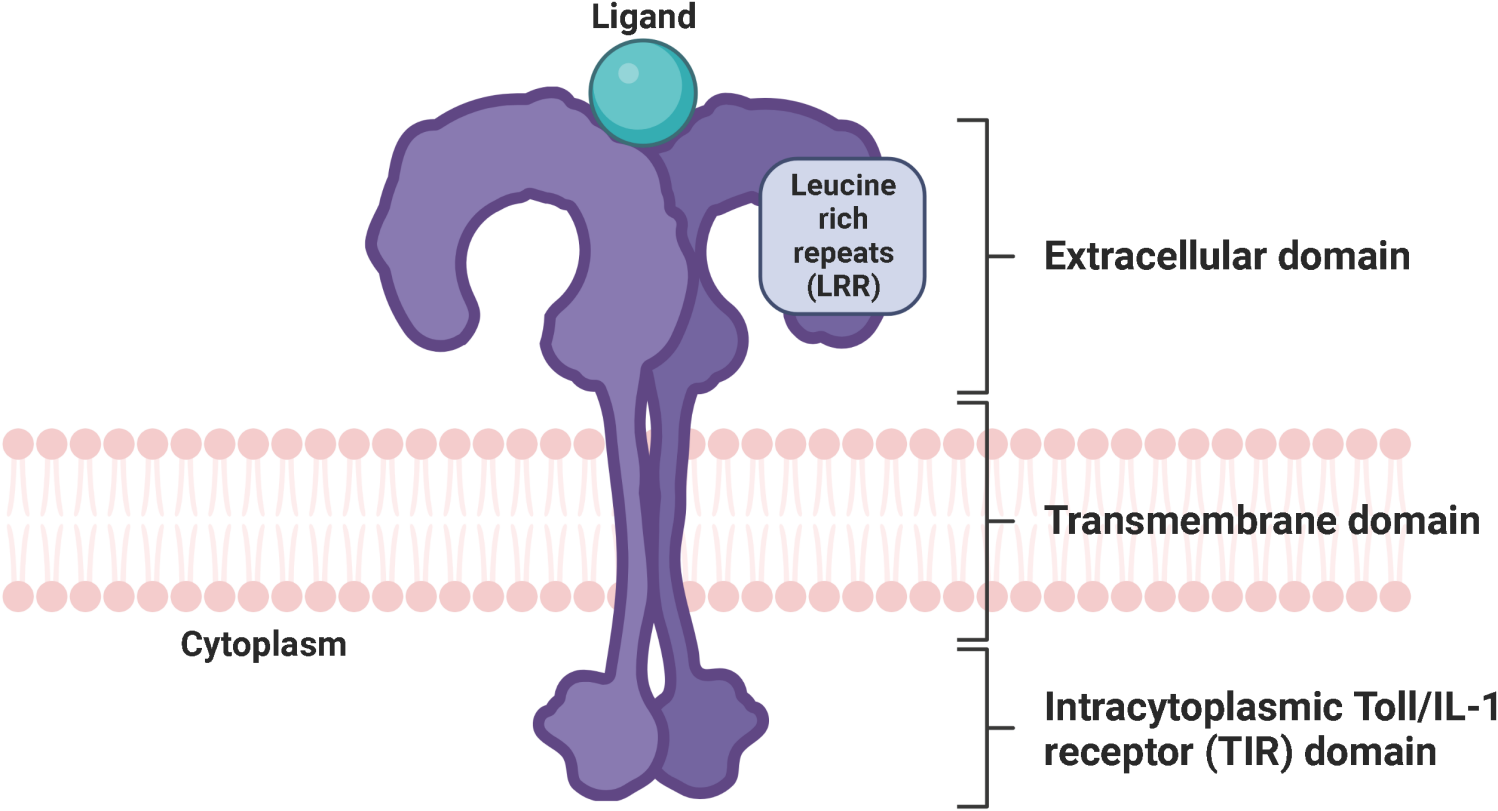
Conserved structure of TLRs. TLRs are characterized by an extracellular domain with leucine-rich repeats responsible for the recognition of PAMPs and DAMPs, a transmembrane domain, and an intracytoplasmic Toll/IL-1 receptor (TIR) domain necessary for signal transduction. TLR, toll-like receptor; LRR, leucine-rich repeats; PAMP, pathogen-associated molecular pattern; DAMP, damage-associated molecular pattern; TIR domain, Toll/interleukin-1 (IL-1) receptor domain. Created with BioRender.com.

Toll-like receptors (TLRs) are a family of conserved pattern-recognition receptors (PRRs) that detect and bind to evolutionarily conserved molecular motifs in pathogen-associated molecular patterns (PAMPs) of microbes or damage-associated molecular patterns (DAMPs) of injured tissues to induce innate and adaptive immune response (1–3). Other classes of PRRs include Nod-like receptors (NLRs), RIG-I-like receptors (RLRs), AIM2-like receptors (ALRs), C-type lectin receptors (CLRs), and intracellular DNA and RNA sensors such as cyclic GMP-AMP synthase (cGAS) (4, 5). Obviously, TLRs are expressed in innate immune cells such as macrophages, neutrophils, dendritic cells, natural killer cells, mast cells, eosinophils, basophils as well as adaptive immune cells, including B cells and specific T cells. They are also found in and on non-immune cells such as fibroblasts, epithelial and endothelial cells (6–8). TLRs are type I transmembrane proteins consisting of an extracellular domain (ECD) with leucine-rich repeats (LRRs) responsible for the detection of PAMPs and DAMPs, a transmembrane region, and an intracytoplasmic toll/interleukin 1 (IL-1) receptor (TIR) domain necessary for the initiation of the downstream signaling pathways (9) (**Figure 1**). LRRs exist in different protein combinations depending on the type of receptor and the corresponding ligand recognition and signal transduction (10). This high variety of LRRs is attributable to its consensus sequence motif of L(X_2_)LXL(X_2_)NXL(X_2_)L(X_7_)L(X_2_), where X can be any amino acid. Upon interaction of distinct PAMPs or DAMPs with the corresponding LRR, the extracellular domains of the respective TLRs form homo- or heterodimers with co-receptors or accessory molecules, which is essential for the activation of various signaling pathways and thus for an appropriate immune response (11). The LRR carboxy-terminal domain, characterized by the consensus motif CXC(X_23_)C(X_17_)C, separates the LRR region from the transmembrane region in TLRs (12). The TIR domain exhibits high homology to that of the IL-1 receptor family (13). Three highly conserved regions in TIR domains have been identified that mediate the protein-protein interactions between TLRs and adaptor proteins necessary for signal transduction. The various TLRs use different sets of adaptor proteins to determine signal transduction, with TLR4 being the only known TLR that uses all TIR domain-containing adaptor proteins, including the myeloid differentiation factor 88 (MyD88), TIR domain-containing adaptor protein (TIRAP), also known as MyD88-adaptor like (Mal), TIR domain-containing adaptor inducing interferon beta (TRIF), and TRIF-related adaptor protein (TRAM) (14, 15).

**Figure 2:**
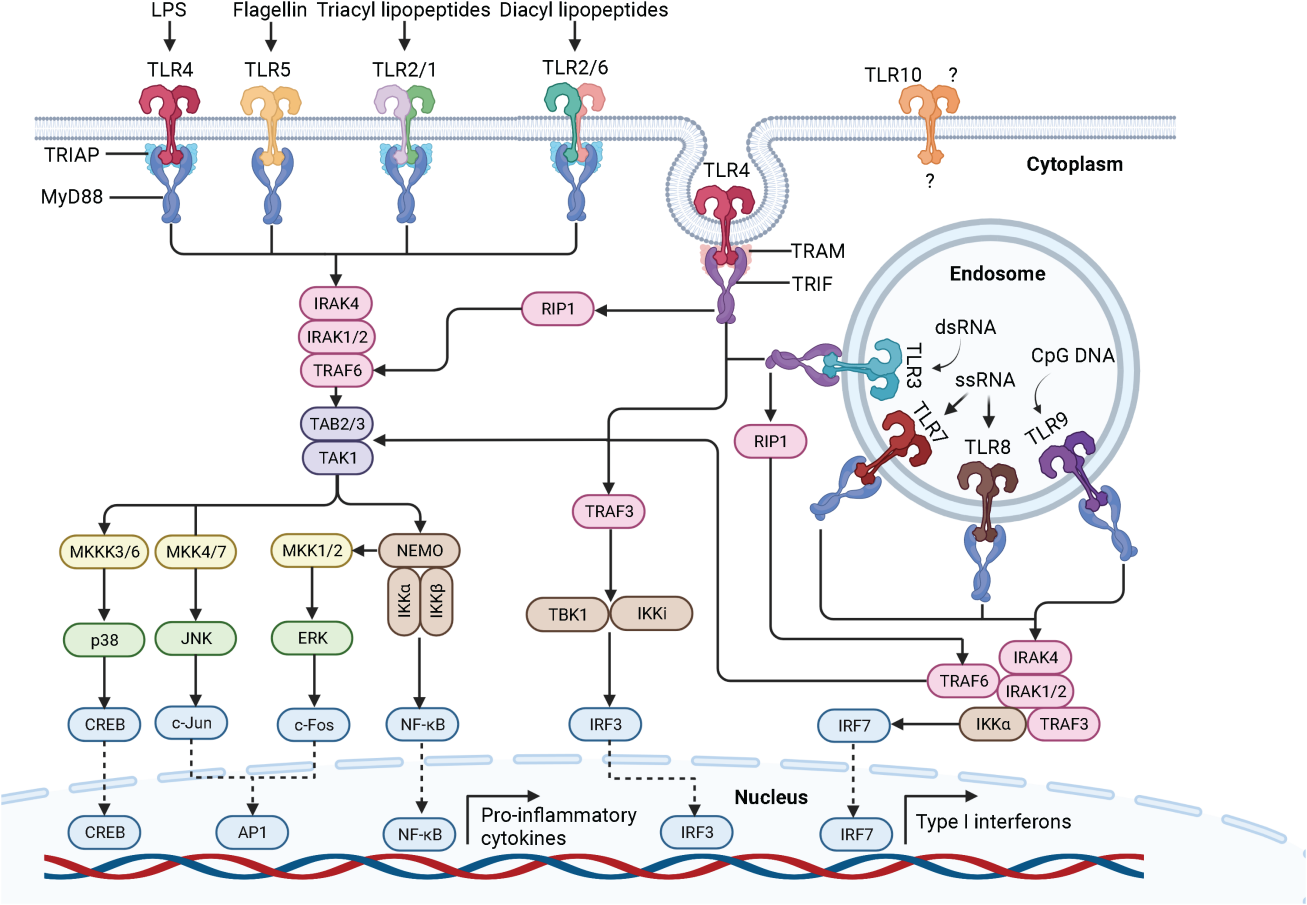
Schematic representation of the TLR signaling pathways. Depending on the type of PAMP and its respective binding affinity to different extracellular domains of the TLRs, TLR signaling is initiated by the dimerization of the extracellular domains of the receptors after recruitment of the adaptor proteins MyD88 and TIRAP (MyD88-dependent pathway) or TRIF and TRAM (TRIF-dependent pathway) through the interactions with the TIR domains of the TLRs. TLR, toll-like receptor; LPS, lipopolysaccharide; dsRNA, double-stranded ribonucleic acid; ssRNA, single-stranded ribonucleic acid; CpG DNA, 5’-cytosine-phosphate-guanine-3’ deoxyribonucleic acid; MyD88, myeloid differentiation factor 88; TRIAP, TIR domain-containing adaptor protein; TRIF, TIR domain-containing adaptor inducing interferon beta; TRAM, TRIF-related adaptor protein; IRAK, interleukin 1 receptor-associated kinase; TRAF, tumor necrosis factor receptor-associated factor; TAK-1, transforming growth factor beta-activated kinase 1; TAB, TAK-1 binding protein; MKK, mitogen-activated protein kinase kinase; JNK, Jun N-terminal kinase; ERK1/2, extracellular signal−regulated protein kinase 1/2; CREB, cyclic adenosine monophosphate-responsive element-binding protein; AP-1, activator protein 1; NF-κB, nuclear factor kappa B; NEMO, NF-κB essential modulator; IKK, IκB kinase; IKKi, inhibitor of NF-κB kinase; RIP1, receptor-interacting protein 1; TBK, TANK-binding kinase; IFN, interferon; IRF, interferon regulatory factor. Created with BioRender.com.

**Figure 3:**
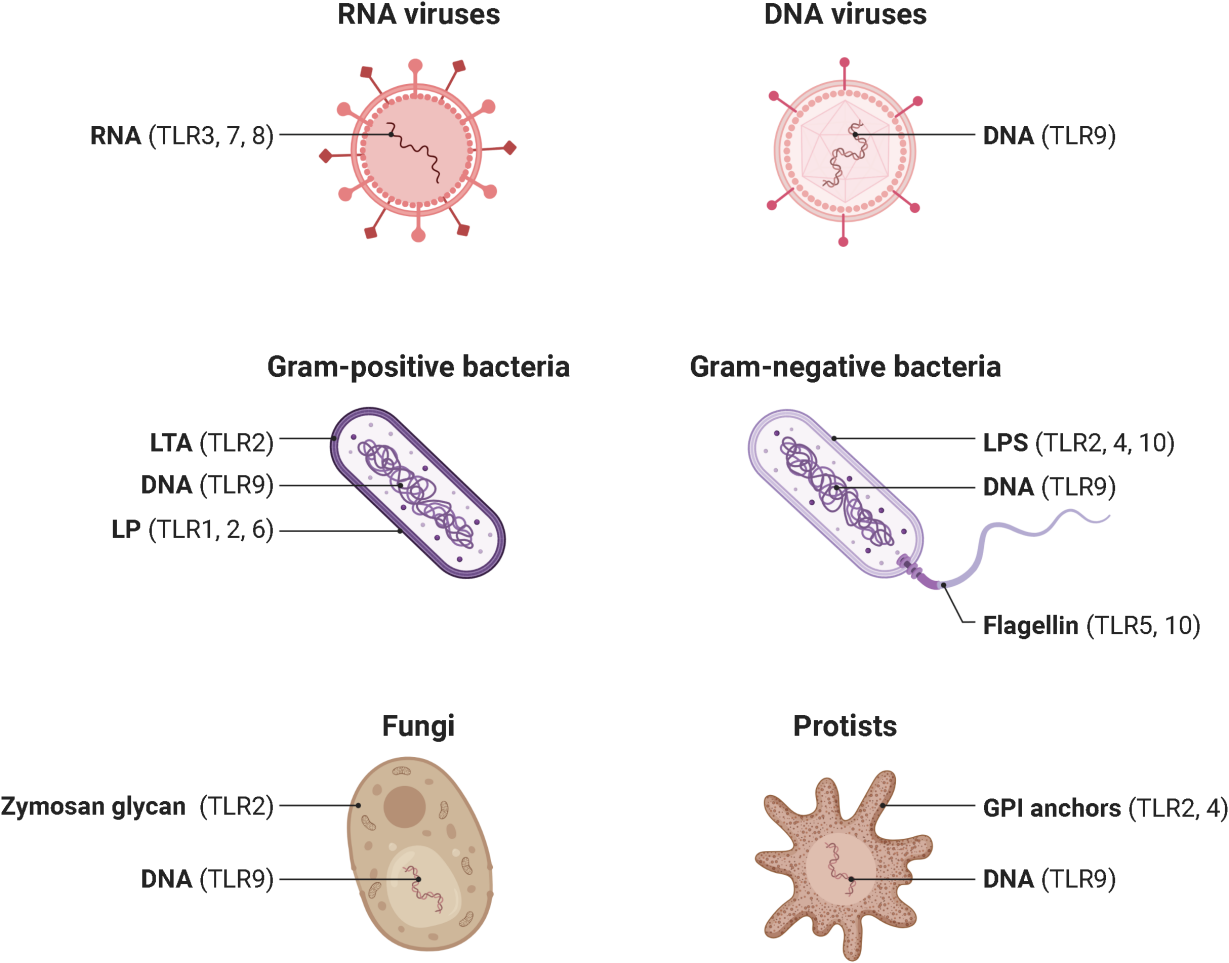
Pathogens and their ligand targets for different human TLRs. Cell surface TLRs primarily recognize components of microbial membranes such as lipoteichoic acid (TLR2), lipopeptides (TLR1, 2, 6) and lipopolysaccharide (TLR4, 10) as well as flagellin (TLR5, 10). Additionally, TLR2 detects zymosan glycan of fungi and, like TLR4, protist GPI anchors. In contrast, cytosolic TLR3, TLR7, TLR8, and TLR9 mainly recognize microbial nucleic acids. TLR, toll-like receptor; DNA, deoxyribonucleic acid; RNA, ribonucleic acid; LTA, lipoteichoic acid. LP, lipopeptides. LPS, lipopolysaccharide. GPI, glycosylphosphatidylinositol. Adapted from “Pathogen Ligand Targets for Different TLRs”, by BioRender.com (2023).

In 1985, Anderson et al. originally identified Toll as a gene product essential for the embryonic dorsoventral axis formation in the fruit fly *Drosophila melanogaster* (16), and 11 years later, its critical antifungal function in this species was described (17). Just one year later, in 1997, the discovery of the mammalian homologue of the toll receptor (now designated TLR4), which plays a major role in the innate immune response by inducing the expression of pro-inflammatory genes, revolutionized our knowledge of the immune system and triggered an irresistible research on PRRs (18). To date, a total of 10 and 12 functional TLRs have been identified in humans and mice, respectively, with TLR1 - TLR9 being conserved in both species. A retroviral insertion into the TLR10 gene in mice impaired its functionality, and the human genome lost TLR11, TLR12, and TLR13 (19). Based on their cellular localization and respective PAMP ligands, human TLRs are classified into cell surface TLRs and intracellular TLRs (**Figure 2**). TLR2/TLR1 and TLR2/TLR6 heterodimers, TLR4 and TLR5 homodimers, and the orphan TLR10 (incomplete understanding of dimerization) are expressed on the cell surface and primarily recognize microbial membrane components such as lipoteichoic acid (LTA) (TLR2), lipopeptides (LP) (TLR1, 2, 6) and lipopolysaccharide (LPS) (TLR4, 10) as well as flagellin (TLR5, 10) (20, 21). In addition, TLR2 detects zymosan glycan in fungi and like TLR4 glycophosphatidylinositol (GPI) anchors in protists. In contrast, human TLR3, TLR7, TLR8, and TLR9 homodimers are exclusively found in intracellular components such as lysosomes, endosomes, endolysosomes, and the endoplasmic reticulum, where they mainly target microbial nucleic acids (9, 22) (**Figure 3**).

## 2 TLR4 signaling

One of the best characterized PAMPs to date is the endotoxin LPS, which is derived from the outer membrane of Gram-negative bacteria. The general structure of LPS is divided into lipid A, a core oligosaccharide, and an O side chain, with lipid A being the major PAMP of LPS (23, 24). LPS stimulation of human cells triggers a series of interactions involving the soluble LPS binding protein (LBP), the soluble glycosylphosphatidylinositol-anchored protein cluster of differentiation 14 (CD14), the soluble myeloid differentiation factor 2 (MD-2), and TLR4 (25, 26). LBP extracts LPS from the outer membrane of Gram-negative bacteria and transports it to CD14, which then transfers LPS to the TLR4/MD-2 receptor complex (14, 27). Interestingly, LPS can bind to MD-2 even in the absence of TLR4, which does not seem to be possible the other way around, as there is only evidence that TLR4 can enhance the binding of LPS to MD-2 (28, 29). Upon LPS recognition, the extracellular domains of TLR4 dimerize and adaptor proteins recruit through interactions with the TIR domains of TLR4. Initial recruitment of the adaptor proteins MyD88 and TRIAP induces the MyD88-dependent signaling pathway, which triggers an early activation of the nuclear factor kappa B (NF-κB) and the mitogen-activated protein kinases (MAPK) (30, 31). Sequential binding of the adaptor proteins TRIF and TRAM and subsequent dynamin-driven endosomal translocation of TLR4 initiates the MyD88-independent pathway, also known as the TRIF-dependent pathway, which culminates in the late-phase activation of NF-κB and the IFN-inducing transcription factor IFN regulatory factor 3 (IRF3). The MyD88-dependent pathway leads to the production of pro-inflammatory cytokines. In contrast, the TRIF-dependent pathway additionally results in the secretion of type I interferons (IFN) (32). The signal transduction of the MyD88-dependent and TRIF-dependent pathway is described in detail in the following sections and is graphically displayed in **Figure 2**.

The MyD88-dependent pathway is characterized by the formation of the Myddosome consisting of MyD88, TRIAP, IRAK4, IRAK1, and IRAK2. MyD88 recruits IL-1 receptor-associated kinase-4 (IRAK4) through homotypic interaction of their death domains (14). IRAK4 is a member of the IRAK family, which shares a death domain and a kinase domain (33). IRAK4 knock-in mutation studies in mice revealed that the kinase activity of IRAK4 is crucial for TLR signaling (34, 35). Of note, IRAK4 also activates NF-κB and MAPK (36). IRAK4 sequentially engages and activates IRAK1 and IRAK2, which are then autophosphorylated at multiple sites (37), leading to the binding of tumor necrosis factor receptor (TNFR)-associated factor-6 (TRAF6) (38). IRAK1 activates the E3 ubiquitin ligase TRAF6, which together with the E2 ubiquitin-conjugating enzymes Ubc13 and Uev1A, induces the synthesis of Lys63 (K63)-linked polyubiquitin to degrade both TRAF6 and IRAK1 and to recruit the transforming growth factor beta (TGF-β)-activated kinase 1 (TAK1) and the adaptor molecules TAK-1 binding protein 2 and 3 (TAB2, TAB3) (11). Subsequent phosphorylation of TAK1 simultaneously induces the IκB kinase (IKK)-NF-κB and MAPK signaling cascades. The IKK complex consists of two catalytic subunits, IKKα and IKKβ, and the regulatory subunit NF-κB essential modulator (NEMO), also called IKKγ. Upon its association with TAK1 through ubiquitin chains, the IKK complex is phosphorylated, leading to the activation of IKKβ (9, 19). Following phosphorylation and proteasomal degradation of the NF-κB inhibitory protein, IκBα releases NF-κB. Released NF-κB then translocates into the nucleus to initiate transcription of pro-inflammatory genes (39). Activated TAK1 simultaneously phosphorylates the MAPK kinases (MMK) 3/6, 4/7, and 1/2, thereby initiating the activation of p38, Jun N-terminal kinases (JNKs), and extracellular signal−regulated protein kinase 1/2 (ERK1/2). Once activated, they phosphorylate the cyclic adenosine monophosphate (cAMP)-responsive element-binding protein (CREB) and activator protein 1 (AP-1) transcription factors, which consist of a heterodimer of c-Fos and c-Jun subunits. The final interaction of CREB and AP-1 with NF-κB facilitates the transcription of pro-inflammatory genes (9, 40).

The TRIF-dependent pathway is characterized by the recruitment of TRAM and TRIF to form the so-called Triffosome (41). TRIF associates with TRAF6 and TRAF3. Activated TRAF6 recruits the kinase receptor-interacting protein 1 (RIP1), which subsequently engages the TAK1 and IKK complex, leading to the activation of NF-κB and MAPKs. Activated TRAF3 recruits the IKK-related kinase TANK-binding kinase 1 (TBK1) and the inhibitor of NF-κB kinase (IKKi), which in turn phosphorylates and activates IRF3, ultimately leading to the induction of IFN (42, 43).

Importantly, once activated, TLR4 is the only member of the TLR family that induces both the MyD88-dependent and TRIF-dependent signaling pathways. TLR2/TLR1, TLR2/TLR6, TLR5, TLR7, TLR8, and TLR9 initiate the MyD88-dependent pathway, whereas TLR3 induces the TRIF-dependent pathway (9, 11). Of note, upon Myddosome formation in response to TLR7, TLR8, or TLR9 activation, IRF7 binds and is directly activated by IRAK1 and IKKα. Like IRF3, IRF7 leads to the induction of IFN (44, 45). We already have a quite clear understanding of the ligand recognition, dimerization, signaling, and biological functions of human TLR1-9, but not of the orphan TLR10. While we know that TLR10 is localized on the cell surface, its ligands, homo- or heterodimerization, TIR domains and adaptor molecules, signal transduction and biological function are still unclear (21).

## 3 TLR4 signaling in endothelial cells under physiological and inflammatory conditions

**Figure 4:**
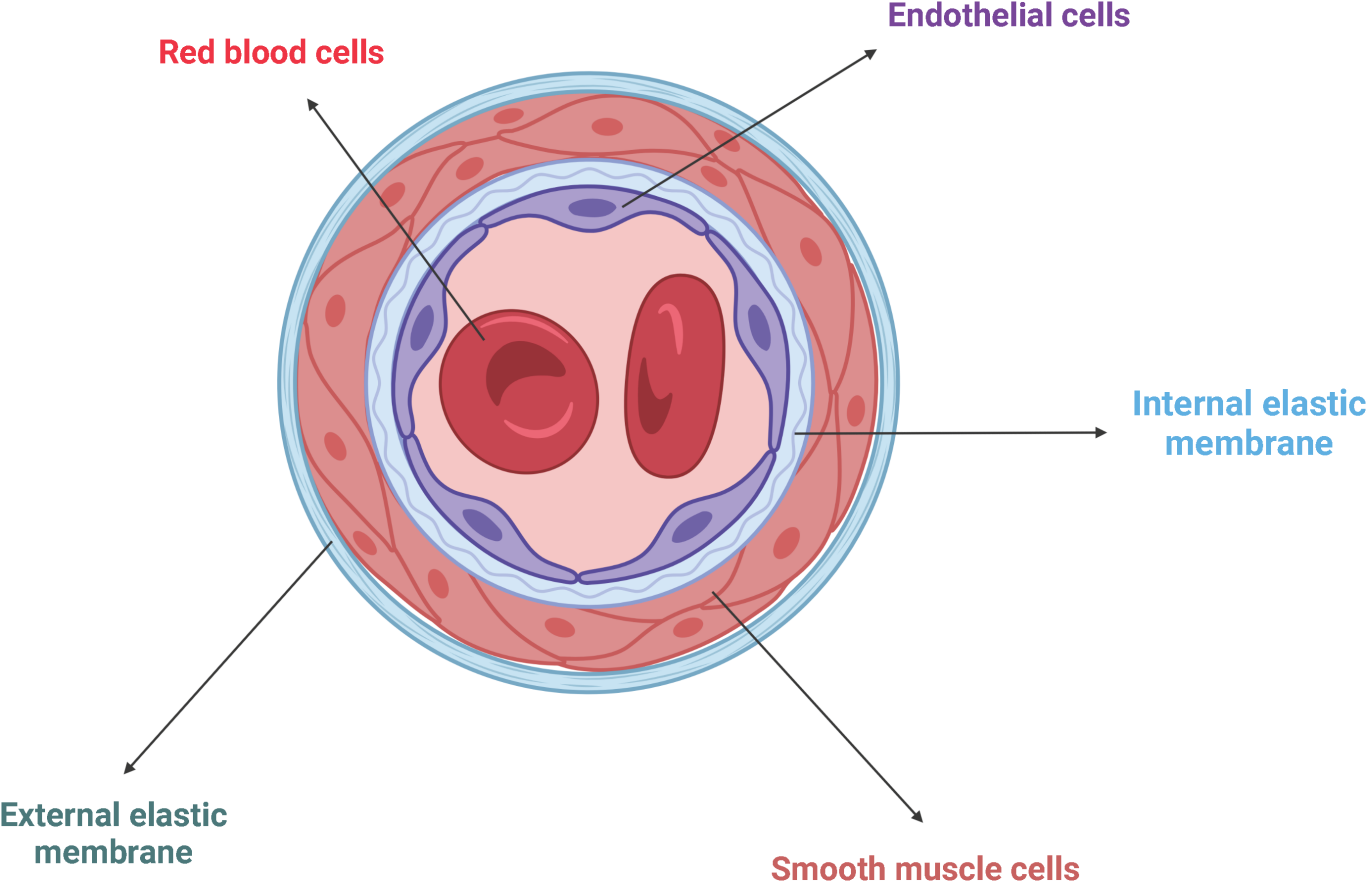
Schematic representation of the general architecture of blood vessels. ECs line the innermost wall of blood vessels, separating the circulating blood from the surrounding tissue. Adjacent smooth muscle cells are trapped between the internal and the external elastic membranes. Created with BioRender.com.

Endothelial cells (ECs) form the innermost layer of blood vessels, which include arteries, veins, and capillaries, and separate the circulating blood from the surrounding tissues (46) (**Figure 4**). Since the blood pressure is much higher in the arteries than in veins, arteries require more layers of smooth muscle cells than veins, making them the thickest blood vessels. Capillaries consist of just one single layer of ECs and no layers of smooth muscle cells because they do not have to withstand any pressure (47).

Due to their unique position, they are the first cells exposed to circulating substances in the blood, under healthy and pathological conditions, and thus act as gatekeepers of vascular health and function (48). Under physiological conditions, the endothelium perform several functions to maintain organ homeostasis, including vasoregulation, selective vascular permeability, and provision of an anti-coagulant environment (49). Upon recognition of PAMPs and DAMPs by PRRs, including TLR1-10, NOD1, and RIG-1, ECs secrete various cytokines, chemokines, growth factors, and adhesion molecules to facilitate vasodilation, promote neutrophil trafficking to the endothelium, activate coagulation pathways, and increase vascular permeability (48, 50–52) (**Table 1**). Of note, studies have highlighted that TLR2, TLR4, and TLR9 are the main drivers of the endothelial immune response (50, 59, 77, 78). In addition to that, by analyzing the baseline mRNA transcription levels of TLR1-10 in different endothelial cell lines, including human umbilical vein endothelial cells (HUVECs), human coronary artery endothelial cells (HCAECs), human microvascular endothelial cells (HMVECs) derived from the brain, liver, and lung, Khakpour et al. clearly demonstrated that TLR4 is the most highly expressed of all TLRs, suggesting that TLR4 is the central PRR in the acute and chronic endothelial immune response (50).

**Table 1:** PRR, especially TLR4-induced responses in ECs.

Pro-inflammatory protein class	Upregulation	Downregulation	Reference
**Cytokines**	IFN-β, IL-1α+β, IL-6, IL-10, IL-18 IL-28, IL-29, IL-33, IL-36, G-CSF, GM-CSF, TNF-α	IL-1RA, IL-10RA, IL-36RA, IL-37	(51, 53–58)
**Chemokines**	CCL2, CCL5, CCL20, CCL21, CXCL8, CXCL9, CXCL10, CXCL11, CXCL12		(54, 55, 57, 59–62)
**Adhesion molecules**	E-selectin, P-selectin, ICAM-1, VCAM-1		(55, 57, 59, 63–67)
**Coagulation factors**	Fibrin, PAI-1, PAI-2, TF, vWF	TFPI, u-PA, t-PA	(52, 68–71)
**Permeability factors**	VEGF	Claudin, Occludin	(51, 72–76)

IFN-β, interferon beta; IL, interleukin; G-CSF, granulocyte colony-stimulating factor; GM-CSF, granulocyte-macrophage colony-stimulating factor; TNF-α, tumor necrosis factor alpha; CCL, chemokine CC-motif chemokine ligand; CXCL, chemokine (C-X-C motif) ligand; ICAM-1, intracellular adhesion molecule-1; VCAM-1, vascular cell adhesion molecule-1; PAI, plasminogen activator inhibitor; TF, tissue factor; vWF, von Willebrand-factor; TFPI, tissue factor pathway inhibitor; u-PA, urokinase-type plasminogen activator; t-PA, tissue-type plasminogen activator; VEGF, vascular endothelial growth factor.

### 3.1 Vasoregulation

To control vascular tone, ECs secrete various paracrine substances. Vasodilators, including nitric oxide (NO), prostacyclin (PGI_2_), and the endothelium-derived hyperpolarizing factor (EDHF), increase the intravascular diameter by relaxing the adjacent smooth muscle cells, whereas vasoconstrictors such as angiotensin, thromboxane (TXA_2_), and endothelin-1 (ET-1), counteract the function of vasodilators to keep a balance in vascular resistance. It is noteworthy that activation of endothelial PRRs, especially TLR4, enhances the expression of vasodilators, leading to decreased blood pressure and increased blood flow, which facilitates endothelial permeability (47, 49, 79).

### 3.2 Leukocyte extravasation/diapedesis/transendothelial migration

**Figure 5:**
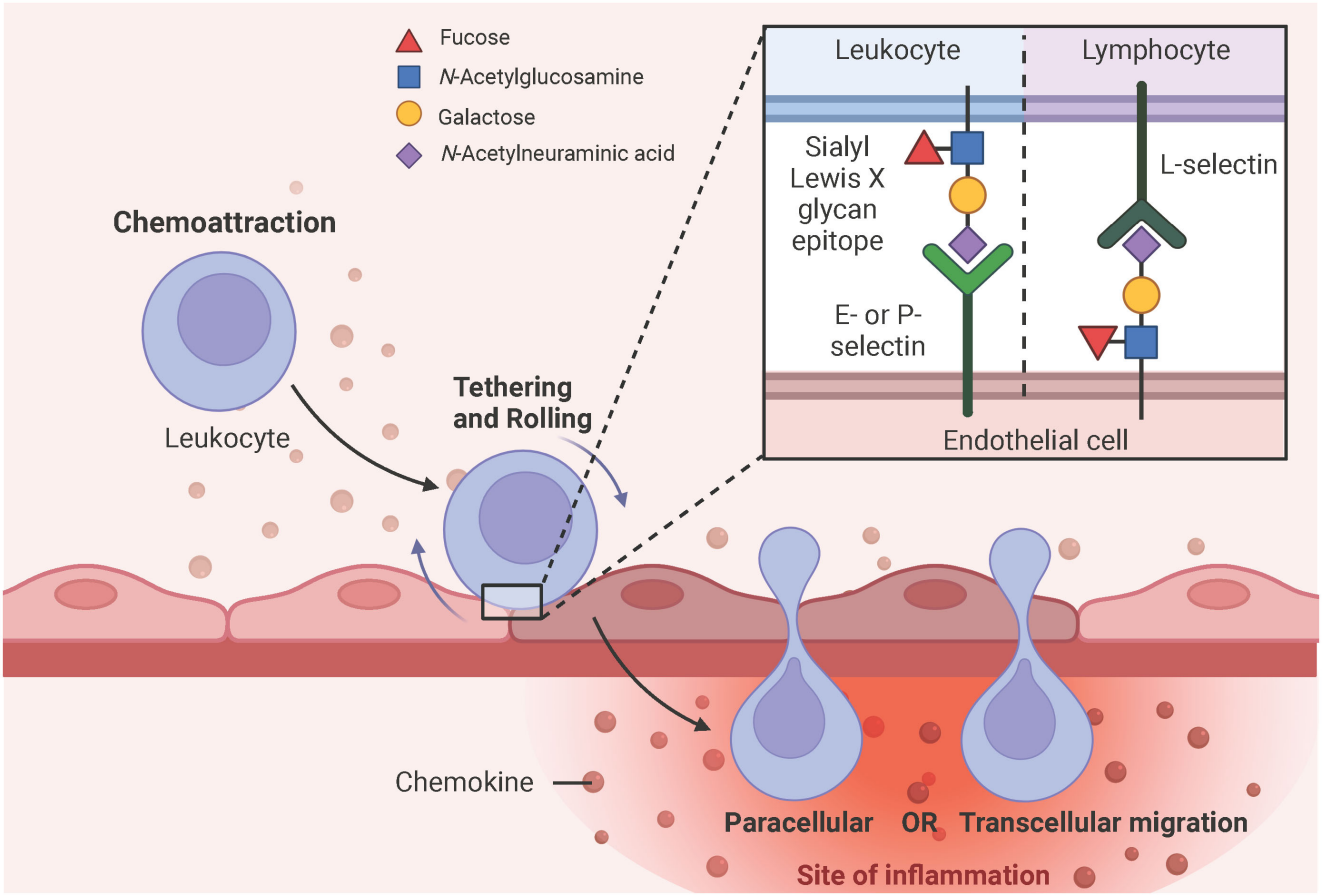
Simplified schematic representation of leukocyte extravasation. PRR, particularly TLR4-induced chemokine secretion initiates the expression of E- and P-selectins on endothelial surfaces, which have a high affinity for sialyl Lewis X glycan epitopes expressed on leukocytes. These selectin-glycan interactions facilitate leukocyte tethering, rolling, and final endothelial transmigration to the site of inflammation. Leukocytes can migrate from the luminal to the abluminal side of the vascular barrier either by the vesicle-based transcellular route through the EC body or by the junctional paracellular route between adjacent ECs. Lymphocytes express L-selectins on their surface and interact with sialyl Lewis X glycan epitopes expressed on ECs to facilitate diapedesis into the subendothelial compartment. Adapted from “Leukocyte Extravasation - The Role of Glycans in Inflammation”, by BioRender.com (2023).

PRR, especially TLR4-induced secreted chemokines, such as CXCL8 and CCL2, initiate the expression of E- and P-selectins at the site of EC activation, which interact with sialyl Lewis X glycan epitopes expressed on leukocytes to tether and roll them (51, 63, 80). Sialyl Lewis X is a tetrasaccharide composed of *N*-acetylneuramic acid (Neu5Ac) α2-3 galactose (Gal) β1-4 [fucose (Fuc) α1-3] *N*-acetylglucosamine and is attached to glycoproteins or glycolipids of cell surface proteins (81–83). Notably, lymphocytes express L-selectin and interact with sialyl Lewis X glycan epitopes expressed on ECs (84). Subsequent deceleration and firm adhesion of rolling leukocytes to the ECs is mediated by β2- and α4-containing integrins expressed on the surface of leukocytes and the intracellular and vascular adhesion molecules (ICAM-1, VCAM-1) upregulated on the surface of ECs in response to the PRR, especially TLR4-induced endothelial cytokine production, such as IL-1 and IL-6 (85–87). Leukocytes then migrate from the luminal to the abluminal side of the vascular barrier either via the vesicle-based transcellular route through the EC body or via the junctional disruptive paracellular route between adjacent ECs (85, 88) (**Figure 5**). Upon arrival at the site of the injured or infected tissue, leukocytes eliminate the necrotic cell debris or pathogens by phagocytosis (50). Of note, neutrophils are the first leukocytes to appear at the site of inflammation and form neutrophil extracellular traps (NETs) to bind, eradicate, and inhibit the distribution of invading pathogens (89).

### 3.3 Coagulation and fibrinolysis

**Figure 6:**
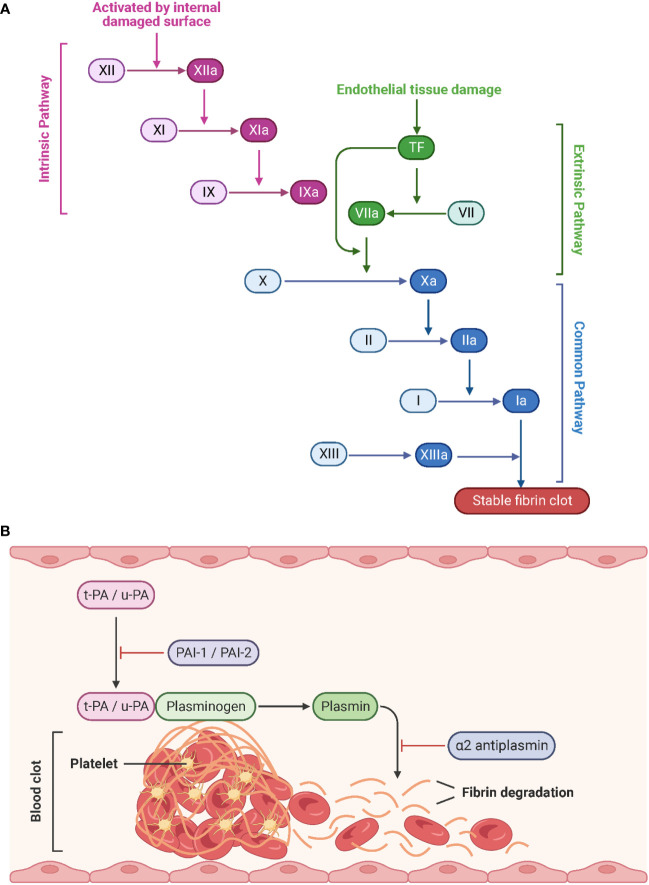
Schematic representation of the coagulation pathways and fibrinolytic pathways. **(A)** Tissue factor (initiation of the extrinsic pathway) and contact activation (initiation of the intrinsic pathway) lead to a common pathway that generates thrombin. Created with BioRender.com. **(B)** During fibrinolysis, t-PA and u-PA convert plasminogen to plasmin, which degrades the fibrin network. Adapted from “Process of Blood Clot Formation”, by BioRender.com (2023).

Generally, the coagulation system can be divided into the fast extrinsic and the slower intrinsic pathway, both of which converge on a common pathway initiated by the activation of factor X to Xa (**Figure 6A**). Tissue factor (TF) and the plasma factor VII are the major initiators of the extrinsic coagulation pathway (90). TF is a membrane-bound glycoprotein that is latent under physiological conditions and released into the blood upon injury. Notably, TF can also be expressed by monocytes and ECs in response to pathogen invasion (91). Factor VII associates with TF to convert to its active form, VIIa. The TF/VIIa complex, in turn, converts circulating factor X to its active serine protease, factor Xa. In contrast, the intrinsic coagulation pathway is initiated by the auto-activation of factor XII to the serine protease factor XIIa upon interaction with negatively charged surfaces such as polyphosphates and phospholipids on activated platelets. This results in the sequential activation of factors XI and IX (92, 93). Thus, both coagulation pathways culminate in the generation of the active factor Xa, which is the starting point of the common pathway. Xa interacts with the cofactor Va to convert prothrombin (factor II) to thrombin (factor IIa). Finally, thrombin cleaves fibrinogen (factor I) to fibrin (factor Ia). The subsequent polymerization of fibrin and aggregation of platelets leads to the formation of a blood clot (94). During the healing process, tissue-type plasminogen activator (t-PA) and urokinase-type plasminogen activator (u-PA) convert the fibrin-bound plasminogen to the active enzyme plasmin, which in turn lyses the fibrin network into a degradable form. Freely circulating t-PA and u-PA are inactivated by the plasminogen activator inhibitor (PAI) 1 and 2, and plasmin is blocked by α2 antiplasmin (**Figure 6B**) (95, 96).

**Figure 7:**
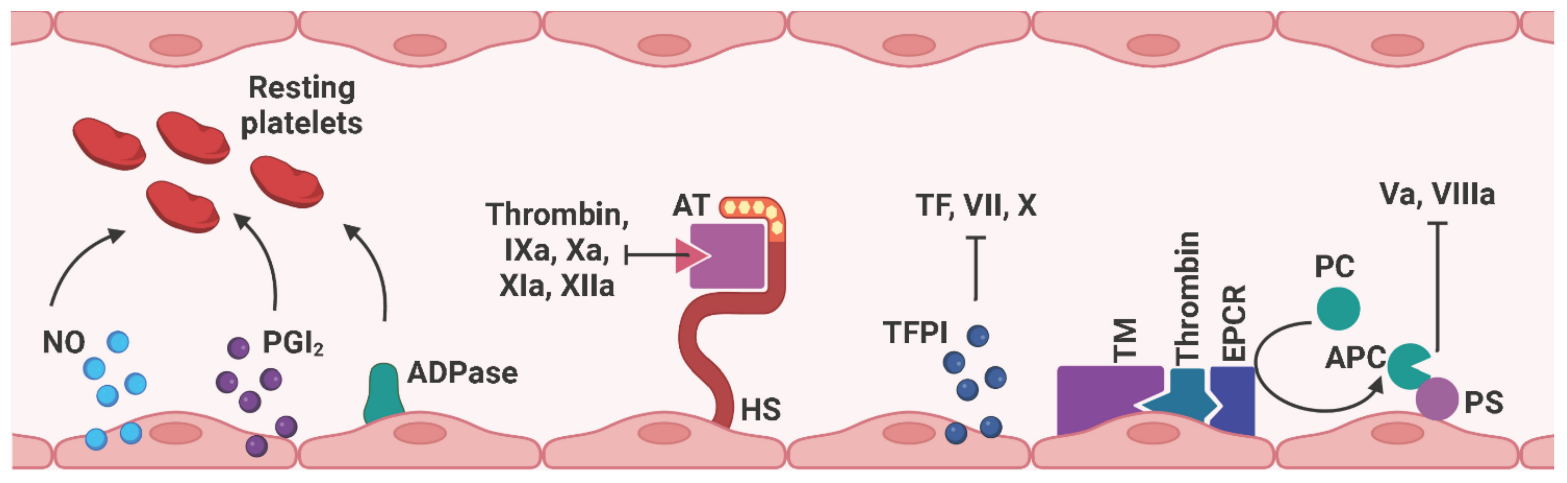
Schematic representation of anti-coagulant mediators expressed on ECs. Under physiological conditions, the endothelium provides an anti-coagulant/anti-thrombotic environment to prevent thrombus formation by free circulating platelets and red blood cells within the blood vessel. NO, PGI_2_, and ADPase prevent platelet adhesion and aggregation. HS cooperates with AT to interfere with thrombin, factors IXa, Xa, XIa, and XIIa. TFPI binds to thrombin, which associates with EPCR, and subsequently released APC interacts with PS to block factors Va and VIIIa. EC, endothelial cell; NO, nitric oxide; PGI_2_, prostacyclin; ADPase, adenosine diphosphatase; AT, antithrombin; HS, heparan sulfate proteoglycan; TM, thrombomodulin; EPCR, endothelial cell protein C receptor; PC, protein C; APC, activated protein C; PS, protein S. Created with BioRender.com.

Since platelets, leukocytes, and erythrocytes circulate continuously in the blood vessels until they are needed, the endothelium must provide an anti-coagulant and anti-thrombogenic environment to prevent platelet adhesion under physiological conditions (97) (**Figure 7**). Therefore, ECs express nitric oxide (NO), prostacyclin (PGI_2_), and adenosine diphosphatase (ADPase) to prevent platelet adhesion and aggregation (93). The glycocalyx is a layer of proteoglycans (PGs) (syndecans and glypicans) and glycosaminoglycan chains (GAGs) (heparan sulfate (HS), chondroitin sulfate (CS) that covers the vascular lumen. Due to its negatively charged composition, the glycocalyx repels platelets and leukocytes from contacting with the ECs (98–101). In particular, heparan sulfate proteoglycans cooperate with antithrombin (AT) to interfere with several coagulation factors, including thrombin, IXa, Xa, XIa, and XIIa (102). Tissue factor pathway inhibitors (TFPIs), as the name suggests, inhibit the coagulation-activating TF, VII, and X. Thrombomodulin (TM) binds to thrombin, which associates with the endothelial cell protein C receptor (EPCR). The subsequently released activated protein C (APC) interacts with protein S (PS) to block coagulation factors Va and VIIIa (103). Notably, Nur77 and Nor1, as well as the inflammatory stimuli C-reactive protein (CRP) and oxidized low-density lipoprotein (oxLDL), have been identified as potential regulators of TM expression and were found to be downregulated in activated ECs, thus exerting a pro-thrombotic effect (104).

**Figure 8:**
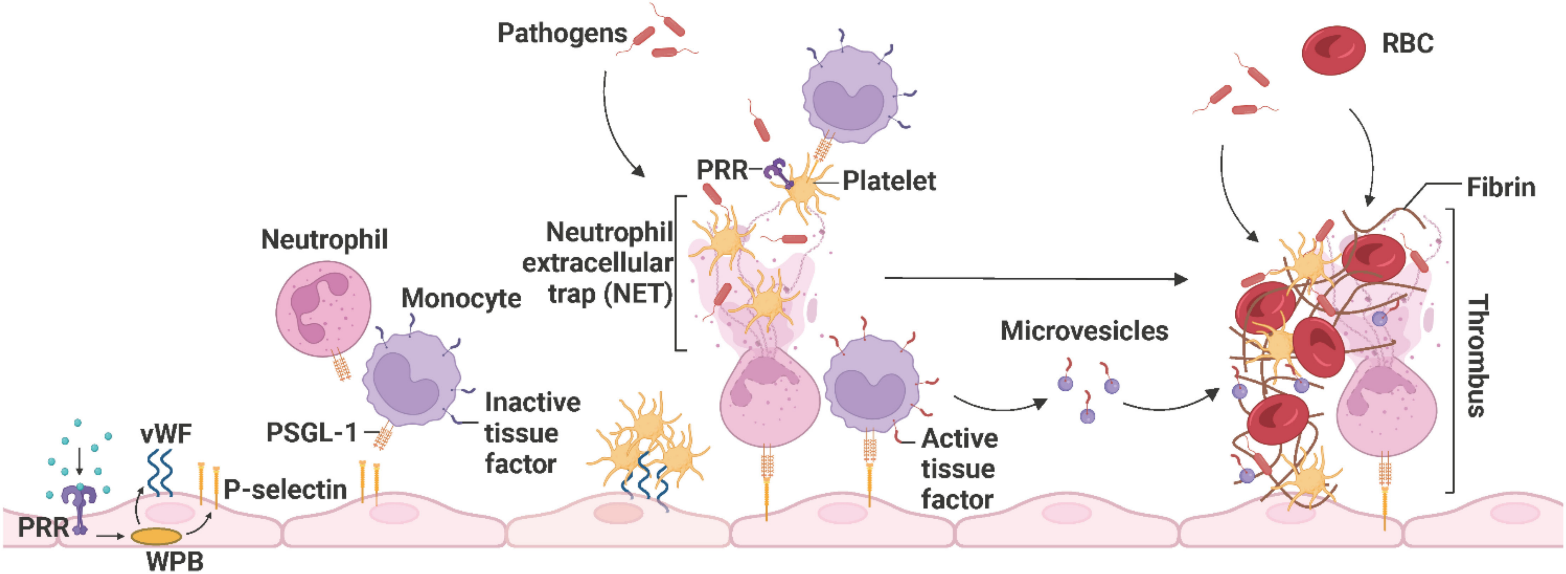
The propagation of immunothrombosis by leukocytes and platelets. During inflammation, PRRs, particularly TLR4, initiate the exocytosis of WPB from ECs and subsequently the expression of P-selectins and vWFs. Leukocytes expressing PSGL-1 are recruited upon interaction with endothelial P-selectin and platelets adhere upon the interaction of endothelial vWF and platelet-derived GPIbα. Neutrophils release NETs that trap pathogens, facilitate thrombus formation, and activate platelets. The activated platelets, recruit leukocytes and recognize pathogens. Active TFs on the surface of monocytes and microvesicles further enhance thrombus propagation by inducing fibrin formation and trapping RBCs. The resulting thrombus promotes pathogen capture. PRR, pathogen recognition receptor; WPB, Weibel-Palade bodies; vWF, von Willebrand factor; PSGL-1, P-selectin glycoprotein ligand-1; NET, neutrophil extracellular trap; TF, tissue factor; RBC, red blood cell. Adapted from “Propagation of Immunothrombosis by Leukocytes and Platelets”, by BioRender.com (2023).

Upon vascular injury or pathogen invasion, the endothelium shifts from the physiological anti-coagulant/anti-thrombotic environment to a pro-coagulant/pro-thrombotic state that promotes fibrin clot formation and reduces clot lysis to prevent blood loss and trap pathogens, respectively (103). This alteration in endothelial function is initiated by sustained activation of ECs by inflammatory stimuli, including circulating PAMPs as described in 1, DAMPs (high-mobility group box 1 (HGMB1), heat shock proteins, heme), cytokines (IL-1β, IL-6, IL-17, IL-19, IFN-γ, TGF-β, TNF-α), chemokines (CXCL1, CXCL8, CCL2), complement system-derived receptors (C1q, C3a or C5a) and proteins (C1, C3, C5, factor B), and reactive oxygen species (ROS) (superoxide anion (O2^−^)) (105–111). Weibel-Palade bodies (WPBs) synthesize and store von Willebrand factor (vWF), P-selectin, and different pro-coagulant and pro-inflammatory proteins. PRR, especially TLR4-induced, exocytosis of WPBs and liberation of their storage components from inflamed ECs leads to the expression of vWF and P-selectin, recruiting platelets and leukocytes such as neutrophils and monocytes, respectively (110, 112, 113). Leukocytes expressing P-selectin glycoprotein ligand-1 (PSGL-1) are recruited upon interaction with endothelial P-selectin, and platelets adhere upon the interaction of endothelial vWF and platelet-derived glycoprotein Ib-alpha (GPIbα) (114). Activated platelets recognize pathogens and further secrete pro-inflammatory and pro-coagulant proteins, including platelet factor 4 (PF4), CXCL4, CXCL5, CXCL8, CCL3, CCL5, and CCL7, which facilitate neutrophil recruitment, tethering, and NET formation. Neutrophils release NETs to capture pathogens, facilitate thrombus formation, and activate platelets. Active TF from the surface of monocytes and microvesicles further enhances the propagation of thrombosis by inducing fibrin formation and trapping red blood cells (**Figure 8**) (68, 115, 116).

### 3.4 Permeability of the endothelial barrier

**Figure 9:**
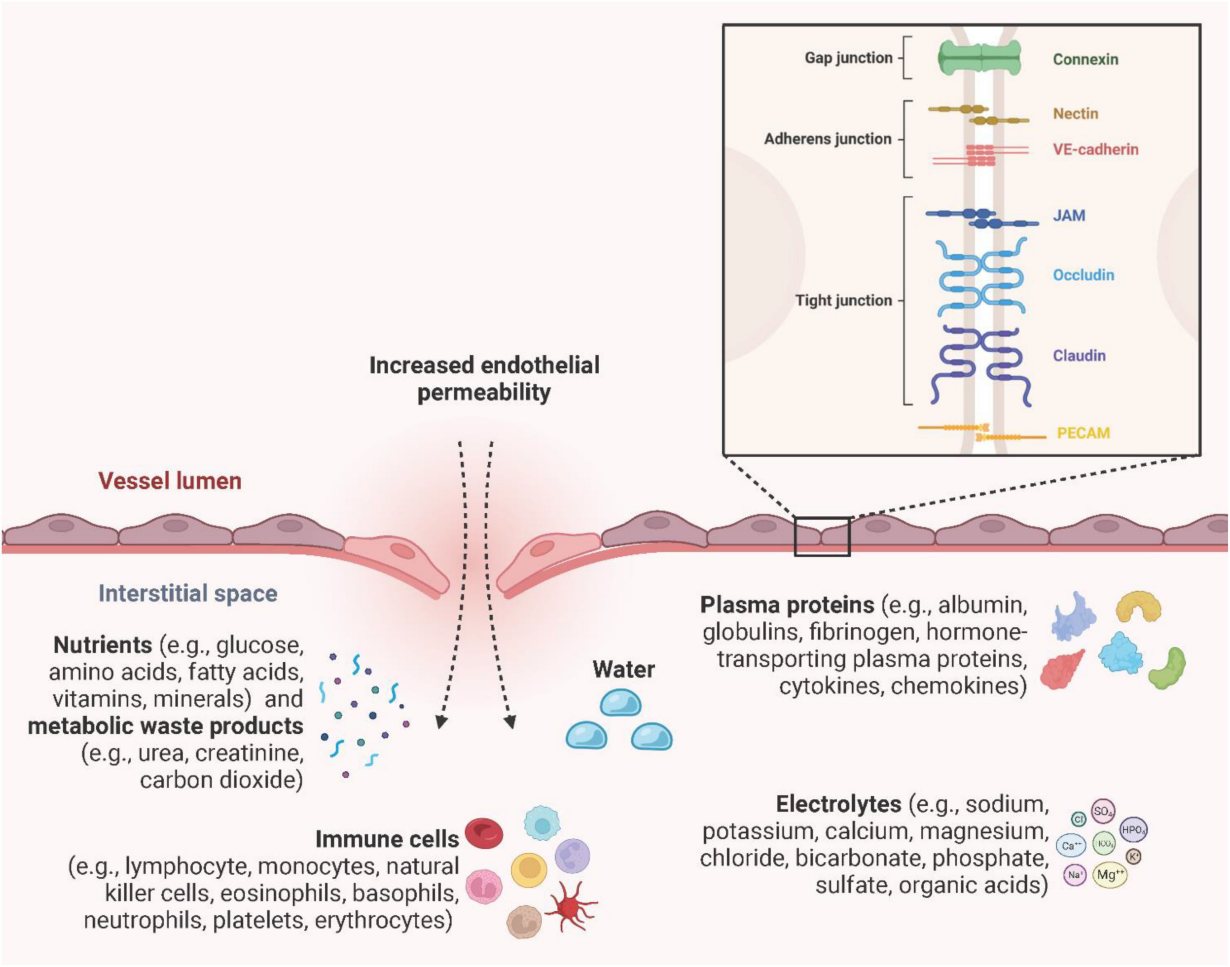
Endothelial permeability. Endothelial integrity and permeability are determined by intracellular junctions, including gap junctions (connexin 32, 37, 40, and 43), adherens junctions (VE-cadherin, nectin), tight junctions (claudin, occludin, JAM A, B, and C), and PECAM-1 to regulate the extravasation of water, plasma proteins, electrolytes, nutrients, metabolic waste products, and immune cells. Created with BioRender.com.

Endothelial integrity and permeability are determined by intracellular junctions between adjacent ECs to regulate the extravasation of water, plasma proteins (e.g., albumin, globulins, fibrinogen, hormone-transporting plasma proteins, cytokines, chemokines), nutrients (e.g., glucose, amino acids, fatty acids, vitamins, minerals), metabolic waste products (e.g., urea, creatinine, carbon dioxide), electrolytes (e.g., sodium, potassium, calcium, magnesium, chloride, bicarbonate, phosphate, sulfate, organic acids), and immune cells (e.g., lymphocytes, monocytes, natural killer cells, erythrocytes, platelets, eosinophils, basophils, neutrophils) (117–119). Inter-EC junctions include tight junctions (claudin, occludin, junction adhesion molecule (JAM) A, B, and C), adherens junctions (VE-cadherin, nectin), gap junctions (connexin 32, 37, 40, and 43), and the platelet-EC adhesion molecule-1 (PECAM-1) (120, 121) (**Figure 9**). Gap junctions are channels that directly link the cytoplasm of adjacent ECs and allow the transmission of electrical impulses, ions, and small molecules to pass between cells (122). Endothelial tight junctions mediate the diffusion of polar solutes and ions and prevent the penetration of macromolecules across the ECs (123). Interestingly, occludins and claudins are indirectly linked to adherens junctions through zonula occludens (ZO) -1, -2, and -3 and further via the actin cytoskeleton (124). Adherens junctions are particularly important in the endothelium, where they stabilize endothelial cell-cell contact and regulate the expression and organization of tight junctions (125, 126). Adherens junctions found in ECs have VE-cadherin as the key transmembrane component and link adjacent cells through its extracellular domain. The cytoplasmic tail associates with p120-catenin through its juxtamembrane domain (JMD) and with β-catenin and plakoglobin through its C-terminal domain (CTD). Plakoglobin or β-catenin are connected to α-catenin, thereby indirectly linking VE-cadherin to the actin cytoskeleton (119, 127). Nectin, a specific member of the endothelial adherens junction family, is a transmembrane protein of the IgG superfamily and enhances homophilic cell-cell adhesion. Nectin sequentially binds afadin, ponsin, α-catenin and vinculin, and finally actin (128). PECAM-1 is a type I transmembrane glycoprotein of the immunoglobulin (Ig) superfamily of cell adhesion molecules and has been found to be highly expressed at inter-EC junctions to maintain barrier integrity by interacting with tight and adherens junctions and acting as a scaffold by engaging β- and γ-catenins (129, 130). PRRs, especially TLR4-induced inflammatory proteins (vascular endothelial growth factor (VEGF), histamine, thrombin, and IL-6) enhance endothelial permeability by activating vasodilators, kinases, and phosphatases to induce augmented actin-myosin contractility, destabilization of the inter-EC junctions and the formation of focal gaps between adjacent ECs (72, 131). Primarily, VEGF, histamine, and thrombin trigger the activation of Src-family tyrosine kinases, which are responsible for the phosphorylation of VE-cadherin, mainly at the tyrosine residue Y685, and its subsequent internalization, thereby strongly promoting endothelial permeability (132). Interestingly, Alsaffar et al. recently demonstrated that the IL-6/Janus kinase (JAK) signaling induces an initial and short-term (2 h) loss of barrier function dependent on Src and MEK/ERK activation, and a sustained permeability requiring signal transducer and activator of transcription 3 (STAT3) phosphorylation at Y705 (131). Transiently increased endothelial permeability in the acute inflammatory response is crucial for tissue repair or pathogen clearance. However, prolonged hyperpermeability leads to pathological conditions such as edema, hypotension, and impaired vascular perfusion and oxygenation of adjacent tissues (127).

## 4 Optogenetics: the art of studying the TLR4 signaling pathway

**Figure 10:**
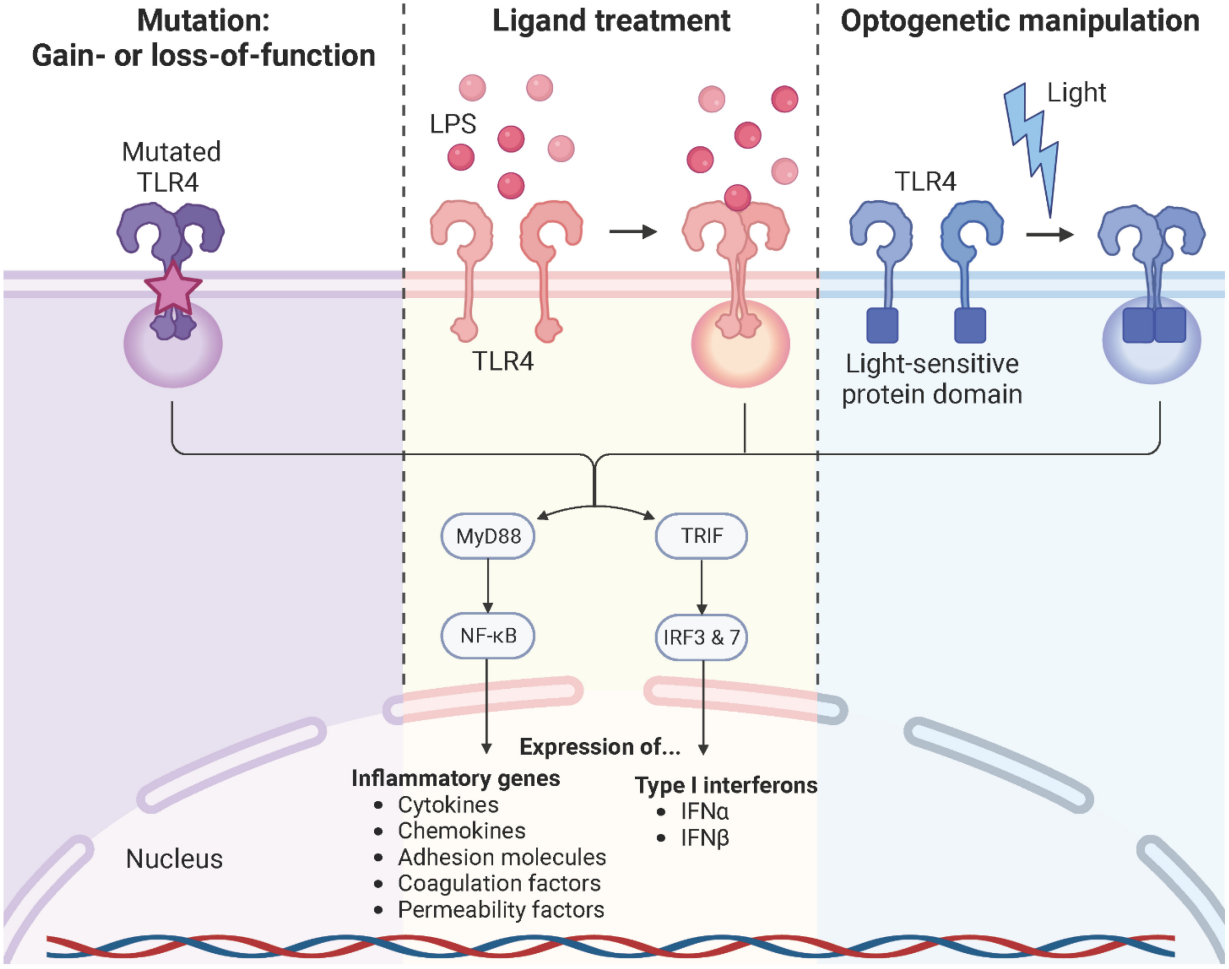
TLR4 modulation strategies. Common approaches used to study TLR4 signaling rely on genetic manipulation through gain- or loss-of-function mutations of the TLR4 or downstream signaling molecules, treatment with its naturally occurring ligand LPS, or optogenetic manipulation. Created with BioRender.com.

TLR4 signaling is a complex, highly dynamic, and tightly regulated network of two distinct pathways that initiate the innate immune response. Persistent TLR4 signaling is responsible for chronic and acute inflammatory disorders, including sepsis (133), atherosclerosis (134, 135), rheumatoid arthritis (136), acute and chronic lung injury (137), sickle cell disease (63, 110, 138), neurodegenerative diseases (139, 140), and cancer (141, 142). For instance, sickle cell disease is a chronic inflammatory condition with hemolysis, vaso-occlusion, and ischemia-reperfusion due to the heme-induced MD-2/TLR4 activation leading to the production of pro-inflammatory mediators and a persistent activation of leukocytes, platelets, and endothelial cells (63, 110, 111, 138). Therefore, modulation of the TLR4 signaling pathway is a promising strategy to specifically target these pathologies. Common approaches used to study TLR4 signaling are primarily based on genetic manipulation through gain or loss-of-function mutations of the TLR4 or treatment with the bacterial endotoxin LPS. However, these strategies are often associated with the generation of irreversible phenotypes in the target cells or unintended cytotoxicity and signaling crosstalk due to off-target or pleiotropic effects. Furthermore, ligands are often unable to penetrate complex tissues, spheroids, or organoids, resulting in surface activation only. On top of that, the use of reagents is associated with complex operational design, high costs, and sources or errors (isolation impurities, batch variations, pipetting errors, instability upon solvation, etc.) (143–145). Here, optogenetics offers an alternative strategy to control and monitor cellular signaling in an unprecedented spatiotemporally precise, dose-dependent, and non-invasive manner (**Figure 10**). It is based on utilizing light-sensitive protein domains of microbial or plant photoreceptors, integrated into effector proteins, to direct them with light stimuli. Hence, light induction allows activation, inactivation, stabilization, destabilization, or localization of signaling pathways depending on the protein type and setup (146–148). Initial optogenetic applications used naturally derived photosensitive opsins to investigate and control neuronal activity and later to study brain circuits. This allowed to replace conventional strategies, most of which were highly invasive, slow in kinetics, and imprecise in targeting specific neurons (149–152). Since then, optogenetics has revolutionized the study of cell biological processes, including signaling pathways, protein movement, or metabolic processes, and was even voted for the Method of the Year 2010 (153, 154). The present available repertoire of light-sensitive domains allows for the formation of protein complexes in response to blue (155–157), red (158, 159), or green (160) light. An important system involves the light oxygen voltage (LOV) domain isolated from the yellow-green algae *Vaucheria frigida*. This xanthophyte contains two distinct aureochromes, aureochrome 1 (AUREO1) and aureochrome 2 (AUREO2), each consisting of a LOV domain and a basic leucine zipper (bZIP) domain. AUREO1 controls blue light-induced cell branching, whereas AUREO2 mediates the development of a sex organ (161, 162). LOV domains are a subfamily of the Per-ARNT-Sim (PAS) family and shear a common PAS domain fold comprising of a five-stranded antiparallel β-sheet and four α-helices (163). The LOV domain derived from the AUREO1 of *Vaucheria frigida* (VfAU1-LOV), noncovalently binds a flavin chromophore that, upon blue light (λmax ≈ 470 nm) absorption, induces a photochemical reaction that leads to the formation of a covalent adduct between the conserved cysteine and the flavin ring. By fusing this blue light-sensing protein domain to the TLR4 and stably incorporating it into endothelial and pancreatic adenocarcinoma cells, Stierschneider et al. developed two physiologically relevant *in vitro* cell culture models in which the TLR4 can be turned on with blue light (470 nm) and turned off in the dark. These newly established optogenetic endothelial and pancreatic adenocarcinoma cell lines allow TLR4-specific studies of the underlying molecular and regulatory mechanisms in inflammation and cancer as well as high content screening for compounds that block TLR4 signaling with spatiotemporal precision (148, 164).

## 5 Conclusion and perspectives

In ECs, activation of TLR4 by LPS ultimately leads to the release of cytokines, chemokines, adhesion molecules, coagulation and permeability factors, that are necessary for an immediate immune response to invading pathogens and tissue injury (12, 165). However, their systemic secretion is a major driver of autoimmune, acute and chronic inflammatory diseases (19). Therefore, negative regulation of TLR4 signaling pathways is a promising strategy to specifically target these pathologies (166). The search for such modulation options requires cell culture models with fast and unambiguous TLR4 signaling. Compared to available standard cell culture models that rely on genetic manipulation of TLR4 or the treatment with agonists such as LPS, optogenetic cell lines with light-inducible TLR4 provide these requirements. Hence, they are predestined for receptor-specific fundamental studies as well as for high content screening of biological active agents that negatively interfere with the TLR4 signaling pathway in inflammation.

## Author contributions

CW: Funding acquisition, Project administration, Supervision, Writing – review & editing. AS: Conceptualization, Visualization, Writing – original draft.
